# Evaluating the mitochondrial function

**DOI:** 10.1186/1758-5996-4-11

**Published:** 2012-04-04

**Authors:** Emin Ozgur Akgul, Mehmet Toygar, Yavuz Poyrazoglu

**Affiliations:** 1 Department of Clinical Chemistry, Gulhane School Of Medicine, Ankara, Turkey; 2 Department of Forensic Medicine, Gulhane School Of Medicine, Ankara, Turkey; 3 Department of General Surgery, Gulhane School Of Medicine, Ankara, Turkey

**Keywords:** Mitochondrial function, Complex activities

## Dear Editor

We have read the article by Xia et.al. entitled as "L-carnitine ameliorated fatty liver in high-calorie diet/STZ induced type 2 diabetic mice by improving mitochondrial function" with great interest [1]. In this article, the authors concluded that L-carnitine might be an effective liver cell protector for delaying the progression of type 2 diabetes mellitus complications. They supported this conclusion by using biochemical and morphological differences between control and L-carnitine-treated groups. However, we have some reservations the terms they used throughout the article: Firstly, the term of "mitochondrial function" has a very broad meaning and might be a little bit confusing as well. As known, mitochondria basically have three biochemical systems: i) β-oxidation, ii) tricarboxylic acid cycle (TCA cycle) and iii) electron transport chain. Although all these systems are interconnected and coexist in mitochondria at the same time, only dysfunctions of electron transport chain are enumerated among the mitochondrial diseases. In this article, we understand that the mitochondrial complex activities were not measured at all. Therefore, we think that the conclusion saying "L-carnitine stimulates fat metabolism" is acceptable. But, using the term of "improvement of mitochondrial function" in this context is not scientifically very appropriate. Secondly, the authors did not describe the method(s) they used in "morphological assessment of hepatic tissue" in detail. They said that tissue samples have been fixed in 4% paraformaldehyde in the third page of the paper. But, as far as we know, oil red O staining works only with fresh frozen tissues. Besides, the red dots in figure 2 were presented as lipid droplets. If these are lipid droplets, then the other unstained vacuoles in the same figure were remained to be identified. We think that the tissues stained with oil red O must be fresh-frozen samples, and at least some of that unstained vacuoles were actually freezing artifacts. Discrimination between freezing artifacts and lipid vacuoles are important because the authors assumed that L-carnitine was decreasing "lipid area percentage" in hepatic tissue.

## Competing interests

The authors declare that they have no competing interests.

## Authors' contributions

Dr. EO AKGUL, MD is the corresponding author of this letter. He evaluated that paper from biochemical aspect. Dr MT, MD is specialist of forensic medicine. He evaluated that paper form morphological aspect. Dr. YP is specialist of general surgery. He drafted the letter. All authors read and approved the final manuscript.
